# Cultural differences in food and shape related attitudes and eating behavior are associated with differences of Body Mass Index in the same food environment: cross-sectional results from the Seafarer Nutrition Study of Kiribati and European seafarers on merchant ships

**DOI:** 10.1186/s40608-018-0180-x

**Published:** 2018-01-24

**Authors:** Joachim Westenhoefer, Robert von Katzler, Hans-Joachim Jensen, Birgit-Christiane Zyriax, Bettina Jagemann, Volker Harth, Marcus Oldenburg

**Affiliations:** 1 Competence Center Health, Department Health Sciences, Hamburg University of Applied Science, Ulmenliet 20, 21033 Hamburg, Germany; 2 Institute for Occupational and Maritime Medicine (ZfAM) Hamburg, University Medical Center Hamburg-Eppendorf, Hamburg, Germany; 3 Preventive Medicine and Nutrition, Institute for Health Services Research in Dermatology and Nursing (IVDP), University Medical Center Hamburg-Eppendorf, Hamburg, Germany; 4 I. Medical Clinic and Polyclinic, University Medical Center Hamburg-Eppendorf, Hamburg, Germany

**Keywords:** Obesity, Pacific islanders, Body shape, Eating behavior, Disinhibition, Seafarer

## Abstract

**Background:**

Overweight and obesity is quite prevalent among seafarers. The present study examined differences in BMI and their association with weight, shape and nutrition related attitudes and perceptions among seafarer from Kiribati, a Pacific Island Group, and European origin.

**Methods:**

The Seafarer Nutrition Study compared 48 Kiribati and 33 European male seafarers from 4 commercial merchant ships. BMI was calculated from measured weight and height. Attitudes to weight, shape and nutrition and disinhibition of control as a characteristic of eating behavior were assessed in a structured interview. Differences between the two groups were examined using t-tests and Chi-square-tests as appropriate. Associations between the variables were examined using Multiple Regression Analysis (MRA) and correlations.

**Results:**

Kiribati seafarer had significantly higher BMI than Europeans (30.3 ± 4.2 vs. 25.6 ± 3.4; *p* < 0.001). However, MRA indicated that Kiribati were choosing thinner shapes as being “most similar” to their appearance than Europeans with the same BMI (B = − 1.14; *p* < 0.05). In addition, Kiribati had significantly higher scores of disinhibition than Europeans (5.6 ± 2.2 vs. 4.3 ± 2.1; *p* < 0.01), and disinhibition correlated with BMI in the Kiribati (*r* = 0.39; p < 0.01), but not in the European group (*r* = 0.17; n.s.).

**Conclusions:**

For Kiribati seafarers the nutrition situation on board represents a highly tempting westernized food environment. Their tendency to disinhibited eating facilitates overconsumption and weight gain, and self-evaluation of their shapes as being thinner than comparable Europeans may hamper appropriate weight control behavior.

## Background

Several studies have shown that overweight, obesity and cardio-metabolic risk factors are quite prevalent among seafarers [1–5].

The situation on board of commercial merchant ships is characterized by limited individual influence on the quality and variety of the diet, because crew members often stay aboard of the vessels for months, and shore leaves are rare and short. Therefore the possibility to buy food is restricted and the nutrition is largely limited to what is offered on board. Likewise, leisure time physical activity is mainly restricted to voluntary exercise in fitness rooms as recreational walking or cycling for longer distances is not possible on board of a ship.

Nevertheless, there seem to be marked differences in the prevalence of obesity and associated risk factors between crew members with different cultural and/or ethnic background working on the same ships and hence in the same environment. A German shipping company reported that particularly crew members from Kiribati, a Pacific island group, experience significant weight gain and possibly impaired cardio-vascular health (personal communication).

This informal observation is in line with the fact that obesity and associated non-communicable diseases, particularly diabetes, present a prevalent and urgent public health problem in the Pacific Region [6]. On Kiribati, 76.5% of adult men are overweight or obese (BMI ≥ 25), among them 39.3% who are obese (BMI ≥ 30). Among adult women 81.8% are overweight or obese and 55.5% are obese [7]. While genetic factors may play an important role for these high prevalences, also cultural factors might be involved in influencing food choices.

Traditionally, large body sizes were positively valued and encouraged in Pacific Island societies. They were associated with wealth, health and beauty [8, 9]. Cortes et al. [8] for example reported that on the Marshall Islands a common greeting has been: “Oh you look good; you look fatter than you did before”. However, these cultural values and perceptions of body size have changed considerably during westernization in the recent decades [9, 10].

A comparison of Samoan and Australian women with similar BMI [11] showed that Samoan woman had similar feelings of fatness as Australian women. However they showed less salience of fatness, felt more attractive and experienced more strength and fitness. While Samoan women had stronger feelings of body disparagement than Australian women, body disparagement was not correlated with BMI in Samoan women whereas is was in Australian women. Brewis et al. [12] reported that Samoan men and women have high levels of obesity, but slim body ideals. However, even when obese they were positive about their body size and weight. A study in the late 1990s reported that both, men and women from the Marshall Islands feel that healthy bodies are larger bodies, whereas thinner bodies are more attractive [8]. More recently, a study found that ratings of attractiveness of women with different BMIs by British and Samoan male adolescents did not differ [13]. The authors concluded that the traditional veneration of large body sizes is not any longer present in the younger generation of Samoan people.

With regard to eating behavior, research has shown that the “disinhibition of control” [14] is consistently associated with weight gain, higher BMI, less healthful food choices and eating disorders like Bulimia nervosa or Binge Eating Disorder [15]. The disinhibition of control reflects the tendency to overeat in response to environmental stimuli (e.g. sight or smell of food, company of others) or emotional stimuli (e.g. anxiety, feeling blue). Therefor we use “disinhibition” and “tendency to overeat” interchangeably in this paper (see methods section). To the best of our knowledge, it has not been investigated whether this characteristic of eating behavior is associated with the high prevalence of obesity in the pacific region.

Therefore the aim of the present study was to examine potential differences in food and shape related attitudes and differences in eating behavior that might contribute to weight gain and obesity in Kiribati seafarers as opposed to seafarers with European origin. Particularly, we assumed that Europeans and Kiribati differed in the relationship between body mass and attitudes to body shape. In addition, we hypothesized that Kiribati could be characterized by a stronger tendency to overeat. And finally, we assumed that being exposed to a western food environment on board as opposed to the traditional Pacific food habits is a tempting situation and prompts the Kiribati seafarers to eat more than at home.

## Methods

### Sample

The 81 seafarers participating in this study came from four cargo vessels belonging to a shipping company with a large number of Kiribati crew members. All explored ships were container ships of similar size (mean 99,400 GT) and had a transatlantic route. The participation rate for this study was 90.9%. Five women and four Africans, as well as one person of Asian origin who were on board of the vessels were excluded from the analysis because we felt that five or less subjects per group was not a sufficient sample size to form a meaningful group for the further analyses of group differences. This resulted in two homogeneous male groups: 48 participants with a Kiribati origin and 33 Europeans.

The participation in this study was voluntary and participants gave their written informed consent. The study protocol was approved by the Ethics committee of Medical Association Hamburg.

### Measures

All measures and interviews were taken on board of the ships. Weight and height of the participants was measured during harbor stays. The interviews were conducted between the 2nd and the 7th day of a transatlantic sea trip during the leisure time of the seafarers. They took place in a separate room with only the interviewer (RvK) and the interviewee present to assure privacy.

Weight and height was measured using standard procedures. Body Mass Index was calculated as BMI = kg/m^2^. A BMI of 25 and more was considered as overweight, a BMI of 30 or more as obese.

**Fig. 1:**
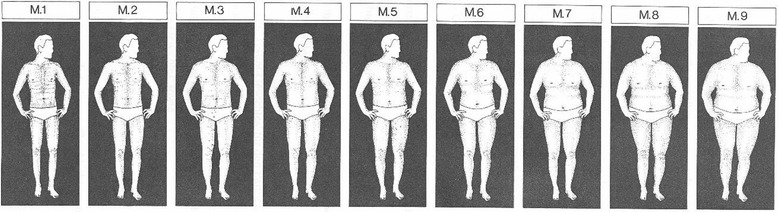
Body shape picture scale. This figure was reproduced from [16] with permission from Deutsche Gesellschaft für Ernährung e.V

Several attitudes regarding weight and shape were assessed in a structured interview with the following questions. Self-evaluation of body weight was assessed with the question “What do you think about your weight? Are you … much too thin – a little bit too thin – just the right weight – a little bit too fat – much too fat”. Answers were scored 1 (much too thin) to 5 (much too fat). Attitudes towards body shape were assessed using a body shape scale [16] which shows 9 different male shapes ranging from thin to obese (see Fig. 1). Subjects were asked to identify the picture that “is most similar to your current shape” (most similar shape), “is most similar to your most desired shape” (most desired shape), “is the most respected shape for a man in your country” (most respected shape), is “the most healthy shape for a man” (most healthy shape). The answers were scored on a rating scale ranging from 1 to 9. If subjects had difficulties to answer the questions on the desired shape and the most respected shape spontaneously, the interviewer explained that “most desired shape” means, what oneself ideally would like to look like oneself, whereas “most respected” refers to what the others in one’s country of origin value most.

The tendency to overeat can be reliably measured with the “disinhibition of control” subscale from the Eating Inventory which often is called Three-Factor Eating Questionnaire TFEQ [14]. This subscale comprises 16 items that address the tendency to overeat in response to environmental stimuli (e.g. sight or smell of food, company of others) or emotional stimuli (e.g. anxiety, feeling blue). It has been originally named “disinhibition of control”, because people trying to restrict their food intake in order to reduce weight or to maintain their weight, so called restrained eaters, often give up their restricted eating behavior in response to such stimuli. From this scale a score is computed that ranges from 0 to 16 with higher scores representing a stronger tendency to overeat. Results related to this facet of eating behavior will be labeled as “disinhibited eating behavior” within the current paper. In order to avoid overly burden for the subjects we used only the disinhibition subscale and omitted the other two subscales (restrained eating and hunger).

In order to collect information about the different views on the nutrition and food situation, a couple of single choice questions were asked in the interview: “Are you satisfied with the supply of food you have on board?”, “Compared to your nutrition at home, do you eat on board … More/Less/To a similar level?”, “Are the foods you receive on board according to your taste?”; “Is the amount of food receiving on your vessel sufficient for you?”; “Does your cook take the preparation of foods for different nationalities into account?” “Is the nutrition on board rich in variety?”

In addition, with regard to the particular food situation on board of the merchant ships, the following open-ended questions were asked in the interview: [1] What do you like most with regard to food and eating on board? [2] Are there things with regard to food and eating that you don’t like or find disturbing? [3] If you compare food and eating with the situation at home: what are you missing most? [4] And again, if you compare food and eating with the situation at home: is there anything that is better on board than at home? The answers to these questions were protocolled verbatim. Using a content analytic approach categories were built by one Author (RvK) [17]: Similar answers were grouped together and a category name was assigned summarizing and abstracting the common content of the answers. The answers were then categorized independently by two raters (RvK and MO). Comparing the results showed an agreement of 95%. Answers without agreement regarding the categorization were excluded from the further analysis.

### Statistical analysis

Statistical analyses were computed using IBM SPSS Version 22.0. Descriptive statistics are reported as mean ± standard deviation. Differences between means were tested using the t-test. Differences in frequency distributions of nominal scale variables were examined using the Chi-square test or Fisher’s exact test were appropriate. Correlations between two variables were examined using Pearson correlation coefficients. The relationship of one dependent variable to two or more (potential) predictor variables was analyzed using multiple regression analysis with all predictors simultaneously entered. Thus, the relationship of the different shape ratings (dependent variables) to BMI, ethnic group and their interaction (predictors) was analyzed using multiple regression analysis with the predictors centered BMI, dummy coded ethnic group (0 = European, 1 = Kiribati) and the product term of the two for the interaction [18, 19]. We used this type of analysis particularly in order to study whether there was an interaction between ethnicity and BMI in predicting the different shape ratings. This would imply that the slope of the relation of BMI to shape ratings is modified by ethnicity. The significance level for all analyses was set to alpha = 0.05.

## Results

The sample consisted of 81 male seafarers, 33 with European and 48 of Kiribati origin. Age did not differ significantly between the European and the Kiribati group (36.8 ± 12.8 resp. 38.9 ± 11.0 years).

### Body Mass Index and Attitudes to Weight and Shape.

Kiribati seafarers and European seafarers differed significantly with respect to BMI (see Table 1). Only 8.3% of the Kiribati were normal weight, 39.6% were overweight but not obese, and 52.1% were obese. In contrast, 48.5% of the Europeans were normal weight, 42.4% overweight but not obese, and only 9.1% were obese.

**Table 1:** BMI, self-evaluation of weight and ratings of the most similar shape, the most desirable shape, most respected shape in the country of origin and most healthy shape: Descriptive statistics (Means ± SD and results from t-tests) and regression coefficients from the multiple regression analysis (MRA) of shape ratings (dependent variables) on ethnic-cultural group (0 = European; 1 = Kiribati), centered BMI and interaction term (predictors)

	Range	Means ± SD		Raw regression coefficients B from multiple regression analysis		
				Predictors for MRAs		
		European	Kiribati	Ethnic Group	BMI	Interaction
BMI		25.6 ± 3,4	30.3 ± 4.2 ^***^			
Self-evaluation of weight	1–5	3.5 ± 0.8	3.5 ± 0.8			
Dependent variables for MRAs						
Most similar shape	1–9	4.3 ± 1.6	4.7 ± 1.8	− 1.14 ^*^	0.34 ^*^	−0.01
Most desirable shape	1–9	3.6 ± 1.1	2.8 ± 1.3 ^*^	− 1.08 ^*^	0.09	− 0.02
Most respected shape	1–9	4.2 ± 1.3	6.7 ± 2.1 ^***^	2.85 ^*^	0.04	−0.17
Most healthy shape	1–9	3.2 ± 1.3	2.5 ± 1.6	−0.44	− 0.06	0.04

^*^ p < 0.05; ^***^ *p* < 0.001

**Fig. 2:**
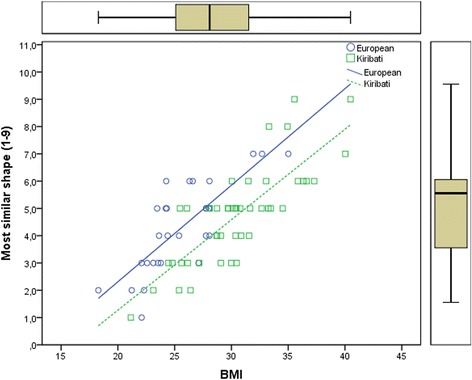
Scatterplot and regression lines of the ratings of the most similar body shape using the body shape picture scale on Body Mass Index BMI

However, both groups did not differ significantly with regard to the self-evaluation of their weight. The mean BMI of the 14 European seafarers who considered themselves as “little bit too fat” or “much too fat” was 28.0 ± 3.3 (range 23.6–35.0), whereas the mean BMI of the 26 Kiribati seafarers who considered themselves as a little or much too fat was 32.5 ± 3.7 (range 25.5–40.5; *p* < 0.01). There was also no significant difference between the two cultural-ethnic groups with regard to the self-evaluation of the “most similar shape” figure. Regression analysis (see Table 1) revealed that the self-evaluation of the “most similar shape” was significantly associated with current BMI. The non-significant interaction indicated that regression slopes in the two cultural-ethnic groups were identical. The significant regression coefficient for the cultural-ethnic group indicated that, on average, Kiribati were choosing the image that was one level smaller than Europeans with the same BMI (see Fig. 2).

The shapes that were selected as the “most healthy” shape were not significantly different between Kiribati and European seafarers, but there were significant differences regarding the “most desirable shape” for one-self and the “most respected shape” in the country of origin. While Kiribati nominated smaller shapes as “most desirable” compared to Europeans, they nominated considerably larger shapes as the “most respected” ones. As a result the difference between the most desirable and the most respected shape was significantly larger (close to three levels) for Kiribati seafarers than for Europeans (*p* < 0.001). Regression analyses showed that the attitudes regarding the most healthy, most desirable and the most respected shapes were not associated with current BMI or with the interaction between BMI and cultural ethnic groups.

### Eating Behavior and attitudes to nutrition and food.

**Fig. 3:**
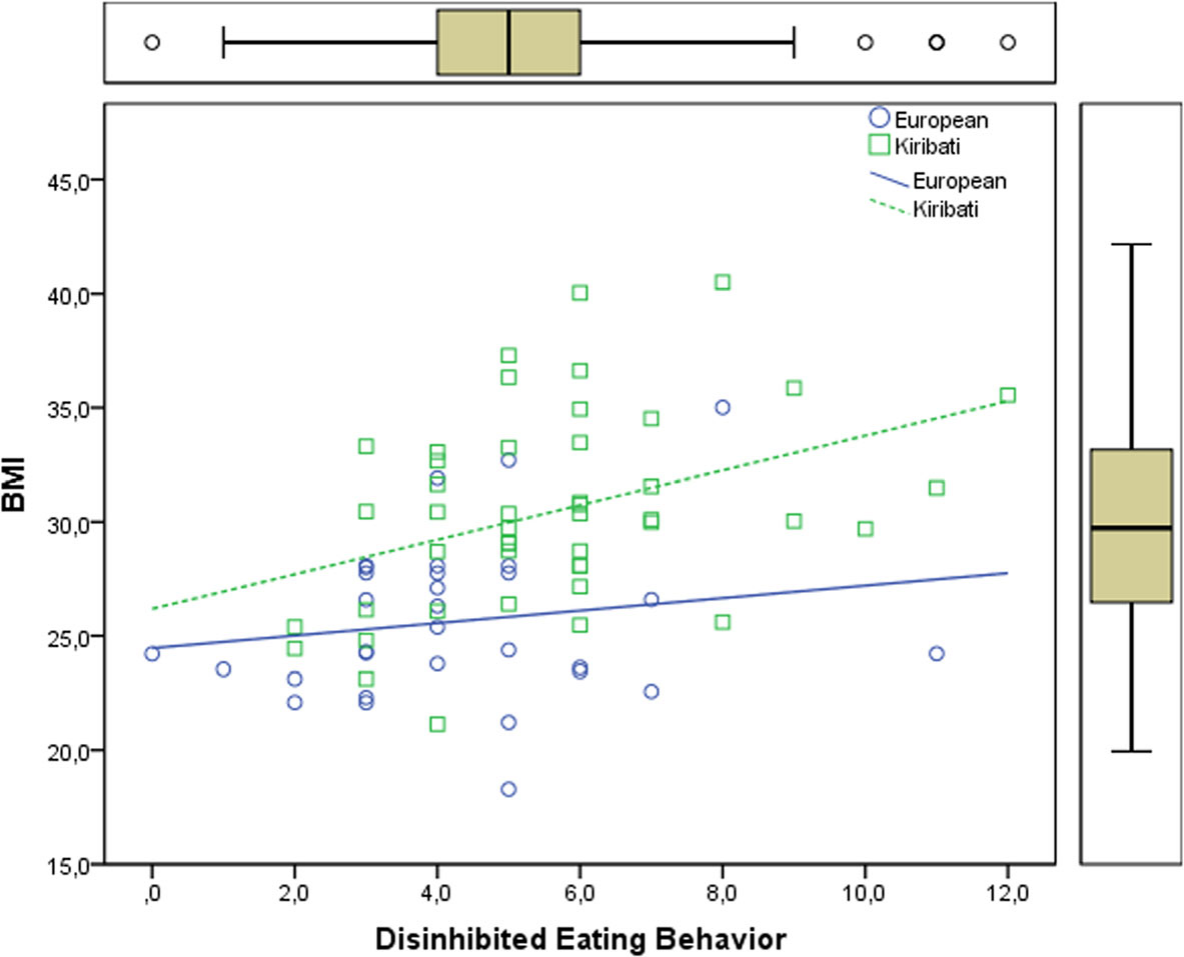
Scatterplot and Regression Lines of Body Mass Index (BMI) on the Scores of the Disinhibited Eating

Kiribati had significantly higher disinhibition scores than Europeans (5.6 ± 2.2 vs. 4.3 ± 2.1; *p* < 0.01). In addition, BMI was significantly correlated with disinhibition in the Kiribati group (*r* = 0.39; p < 0.01), but not in the European group (*r* = 0.17; n.s.) (see Fig. 3).

**Fig. 4:**
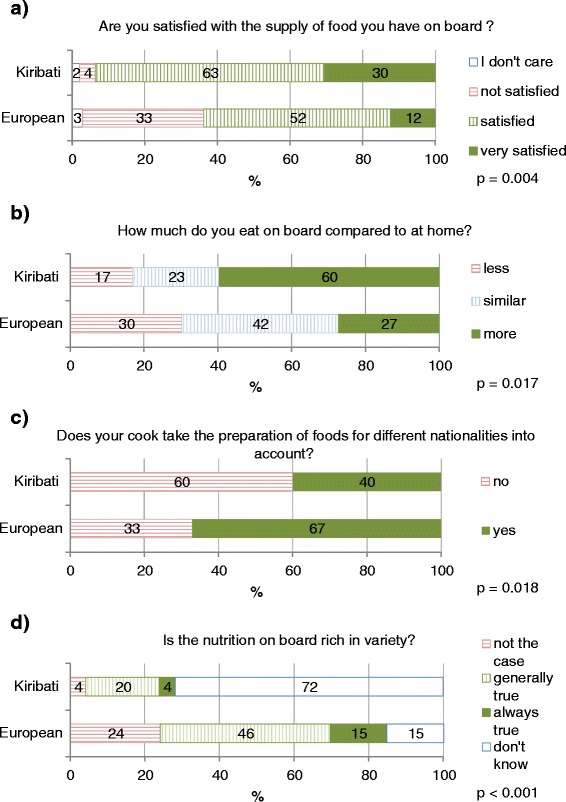
Evaluation of food and nutrition situation on board of commercial merchant ships by European and Kiribati seafarers. **a** Are you satisfied with the supply of food you have on board? **b** How much do you eat on board compared to at home? **c** Does your cook take the preparation of foods for different nationalities into. **d** Is the nutrition on board rich in variety? account?

Kiribati seafarers reported significantly more often than Europeans to be satisfied with the food supply on board and to eat more on board than at home (Fig. 4). However, there were no significant differences between the two groups with regard to the tastiness of the meals (*p* = 0.077) and whether the amount of food is acceptable (*p* = 0.65; data not shown). Furthermore, Kiribati and Europeans differed significantly in their opinion whether the cooks prepare special meals for the different nations on board and in their appraisal of the variety of the food in the meals (Fig. 4). Particularly striking is the high portion of Kiribati who state that they don’t know whether meals include a variety of foods.

**Fig. 5:**
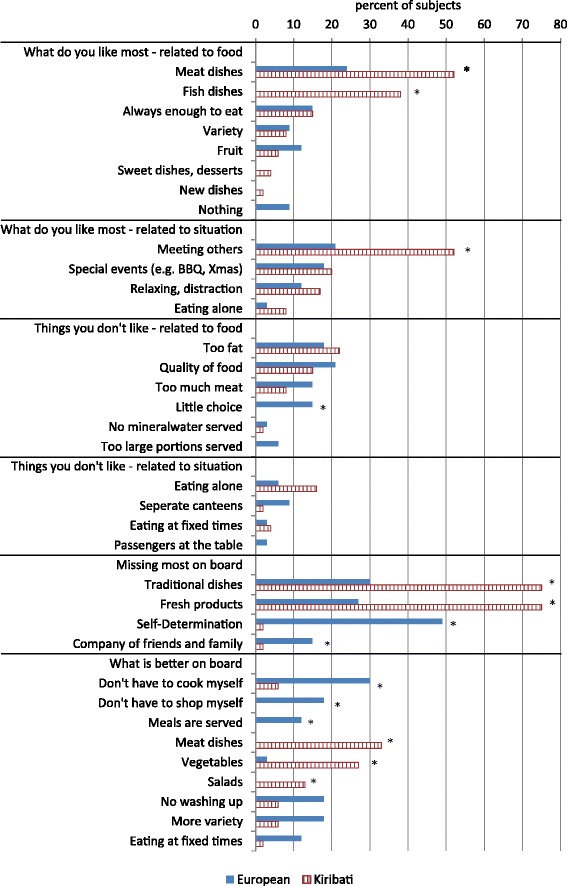
Percentage of categorized answers to open questions about food and eating on board of commercial merchant ships. Significant differences (*p* < 0.05) between European and Kiribati seafarers are marked with *

The relative frequency of the categorized answers to the open questions regarding food and eating on board is shown in Fig. 5. Kiribati mentioned significantly more often than Europeans meat and fish dishes as things they like most. Correspondingly, also one third of the Kiribati, but none of the Europeans mentioned meat dishes as being better on board than at home. In addition, Kiribati mentioned more often meeting with others during meals as something they appreciate. Three quarters of the Kiribati stated that they were missing traditional dishes and fresh products on board, whereas Europeans were missing self-determination and the company of friends and family more often. Last, not least, Europeans appreciated several aspects of convenience of the eating situation considerably more often than Kiribati.

## Discussion

Despite living, working and eating in the same food environment for most of the time, namely on commercial merchant ships, there are marked differences in Body Mass Index between Kiribati and European seafarers. The present study examined attitudes and perceptions related to weight, shape, eating and food for differences between the two cultural-ethnic groups and their association to the Body Mass Index.

In summary, the present study found that despite being considerably heavier than Europeans Kiribati perceive their weight and shape as being on the same level as the Europeans. When selecting pictures from the Body Shape Scale representing their appearance, on average they select thinner shapes than Europeans with the same BMI. Likewise, Europeans considering themselves as being “too fat” have significantly lower BMIs than Kiribati considering themselves as being “too fat”. Compared to Europeans, Kiribati nominate thinner body shapes as “most desirable” for a man, but larger shapes as the “most respected” shape in their home country. The question on the “most desired shape” was intended to elicit the view about what the subject himself perceives as an ideal shape for himself, whereas the question of the “most respected shape” was intended to elicit the subjects view about social norms in his country of origin. The difference in the answers to the two questions indicates that the subjects indeed recognized these different perspectives. Thus, the present findings about the “desirable” shape are in line with results from other pacific islands that found that both men and women attributed attractiveness to thinner bodies [8]. The views of Kiribati on the most respected shape reflect the more traditional veneration of large body size in Pacific populations. Such valuation with regard to men seems to persist to a certain degree at least in adult males. This observation questions the idea that such veneration is not any longer present [13].

The higher BMI of Kiribati seafarers corresponds with a significantly higher level of disinhibited eating which reflects the tendency to overeat in response to environmental and emotional stimuli. In addition, the disinhibition of eating behavior correlates with BMI in the Kiribati group, but not in the European group.

The tendency to overeat in response to environmental stimuli could be particularly important, given the finding that the majority of Kiribati reports that they eat more on board than at home, and that they are more satisfied with the nutrition situation on board of the vessels than the Europeans. Particularly, the Kiribati enjoy the availability of meat dishes and fish and they value the social company with others during meals more than the Europeans. Thus, overall it seems that Kiribati experience the food and nutrition environment on board very positive, and this might be potentially stimulating appetite. However, the vast majority of Kiribati is missing traditional dishes and fresh products, and they feel more often than Europeans that food preparation does not take differences between nationalities into account. Hence, Kiribati seem to experience the nutrition situation on board as foreign, but overall quite positive and appetizing for them. This in turn might predispose them to overeating and weight gain.

In addition, differences in the social environment on board from the situation at home could contribute to differences in eating behavior and food intake. Kiribati are considered to be a sociable population group who appreciate meals together with others as an important element of their cultural tradition [20]. The average household has 7 or more members and common meals at home play an essential role in social relationships and familial tradition [21]. In contrast, meals on board are offered in the crew’s messroom in immediate vicinity of the working place. Mealtimes are entirely determined by work requirements. Meals are nearly always eaten together with other crew members who are determined by working conditions, not by familial relations. Preparing own meals is impossible.

In contrast, European seafarers value particularly the convenience aspects of the nutrition situation (being served, no need of cooking, shopping, washing up), but are more unsatisfied with the lack of self-determination regarding food and the restricted choice they have on board. These negative experiences are nearly unmentioned by the Kiribati. Taken together, the findings from the present study suggest that the more western food environment on commercial merchant ships prompts Kiribati seafarers to eat more than at home, enjoying particularly meals with meat. This environment could trigger higher food intakes particularly in view of the higher responsiveness of Kiribati to such environmental stimuli.

The views of a healthy body shape, and presumably weight, are not much different for Kiribati and Europeans; the desirable shape is even thinner for the Kiribati. However, given the cultural valuation of the larger body shape in Kiribati, they perceive themselves as thinner as Europeans with the same BMI. Such differences in self-evaluation could prevent appropriate adjustment of eating and activity behavior in order to prevent weight gain.

The present study has some underlying limitations which have to be considered when interpreting results and drawing conclusions. First of all, we studied a relatively small sample of 33 European and 48 Kiribati male seafarers. Hence, findings and conclusions have inherent uncertainty. However, this sample represented more than 80% of the total crew of 4 ships and can be considered as highly representative for European and Kiribati crew members. Moreover, our findings are clearly limited to males. However, since most of the research on body image is done in women, the present study adds some insights on the cultural differences between men from European and Oceanian origin. Secondly, the socio-economic position of European and Kiribati crew members is not entirely comparable, because European crew members hold more often higher positions on board. However, this cannot be balanced as it reflects the current reality on board of commercial ships. Third, the present study is cross-sectional and thus precludes any causal interpretations. Forth, our study design does not allow to distinguish between socio-cultural and biological-genetic factors. Thus, we cannot exclude that some differences and associations which we interpreted as cultural differences are at least partially associated with genetic differences.

## Conclusions

Having these limitations in mind, our study suggests that men from Kiribati who hire as seamen on commercial merchant ships are more susceptible to overeating in response to environmental stimuli and perceive themselves as thinner than Europeans. For them the nutrition situation on boards represents a highly tempting westernized food environment which facilitates overconsumption and weight gain.

Thus, the present study and its results appear to be small scale model of the much broader problem of the globalization of western influences. On board, food insecurity is no problem: Physical and economic access to food is simply given. Food is provided at little cost, actually for free, in a regular, predictable manner, in amounts that are more than sufficient. Food supply includes foods which are highly valued, particularly meat, and therefore tempting. All of these elements are relatively new for people living in or coming from food environments which have been traditionally characterized by scarcity of food. In parallel, such food scarce environments have traditionally established the ideal of large body sizes [8, 9]. When globalization offers the opportunities to have at little costs and efforts what long has been desired, it is not surprising that people use these opportunities to overeat. Traditional social values may justify to a certain degree weight gain, even though they might be in conflict with individual ideals which in turn are changing to approach the western standard of slenderness. The heightened tendency to overeat has been associated with body mass index of Kiribati in the present study. This is consistent with the body of evidence [15]. However, an interesting question for further research is whether the higher tendency to overeat is a consequence of deprived subjects being exposed to an environment that provides the deprived objects. Experimental research on chocolate “deprivation” suggest that such mechanisms could play a role [22].

Further research should explore the potential of changing some elements of the environment on board to improve food choice and eventually weight development of the seafarers. An obvious option would be the provision of information about healthy food choice directly at the table. However, given the limited influence of nutrition knowledge on eating behavior, this might not be sufficient. A promising option would be to apply the principles of nudging [23], e.g. by offer of fresh fruit and vegetables as first courses of the meals, because research has shown that the order of presentation of food influences food choice and consumption [24]. Additionally, adding more choice options during meals would probably increase satisfaction with the eating situation, particularly the needs of European seafarers for more self-determination.

On board of merchant ships is an interesting research environment, because external influences are rather limited to very short periods of port visits. Thus, ships could represent an ideal “laboratory” environment to study the influence of environmental changes on eating behavior and weight development. Further research should exploit this potential. Particularly, Kiribati seafarers who are susceptible to weight gain, overweight and obesity would directly benefit from the development of environmental measures to encourage adequate energy intakes while maintaining the satisfying features of on board nutrition.

## Abbreviations

BMI: Body Mass Index
GT: Gross Tonnage
MRA: Multiple Regression Analysis
n.s.: not significant
resp.: respectively
SD: Standard Deviation
TFEQ: Three factor eating questionnaire
vs.: versus
